# Inflammasomes and Fibrosis

**DOI:** 10.3389/fimmu.2021.643149

**Published:** 2021-06-11

**Authors:** Wen-Juan Zhang, Shu-Juan Chen, Shun-Chang Zhou, Su-Zhen Wu, Hui Wang

**Affiliations:** ^1^ Department of Immunology, School of Basic Medicine, Gannan Medical University, Ganzhou, China; ^2^ Key Laboratory of Prevention and Treatment of Cardiovascular and Cerebrovascular Diseases of Ministry of Education, Gannan Medical University, Ganzhou, China; ^3^ Department of Experimental Animals, Tongji Medical College, Huazhong University of Science and Technology, Wuhan, China; ^4^ Department of Biochemistry and Molecular Biology, School of Basic Medicine, Gannan Medical University, Ganzhou, China; ^5^ Henan Key Laboratory of Immunology and Targeted Drugs, Xinxiang Medical University, Xinxiang, China

**Keywords:** inflammasome, fibrosis, NLRP3, caspase-1, IL-1β

## Abstract

Fibrosis is the final common pathway of inflammatory diseases in various organs. The inflammasomes play an important role in the progression of fibrosis as innate immune receptors. There are four main members of the inflammasomes, such as NOD-like receptor protein 1 (NLRP1), NOD-like receptor protein 3 (NLRP3), NOD-like receptor C4 (NLRC4), and absent in melanoma 2 (AIM2), among which NLRP3 inflammasome is the most studied. NLRP3 inflammasome is typically composed of NLRP3, ASC and pro-caspase-1. The activation of inflammasome involves both “classical” and “non-classical” pathways and the former pathway is better understood. The “classical” activation pathway of inflammasome is that the backbone protein is activated by endogenous/exogenous stimulation, leading to inflammasome assembly. After the formation of “classic” inflammasome, pro-caspase-1 could self-activate. Caspase-1 cleaves cytokine precursors into mature cytokines, which are secreted extracellularly. At present, the “non-classical” activation pathway of inflammasome has not formed a unified model for activation process. This article reviews the role of NLRP1, NLRP3, NLRC4, AIM2 inflammasome, Caspase-1, IL-1β, IL-18 and IL-33 in the fibrogenesis.

## Introduction

Fibrosis is a common stage in the progression of various organ inflammatory diseases. Its “typical” feature is the deposition of collagen and the formation of extracellular matrix (ECM) (1). The common pathological process of fibrogenesis is that after endogenous/exogenous factors damage organs, macrophages (Mø) in organs activate and release a large number of cytokines, such as transforming growth factor-β (TGF-β) and interleukin-1β (IL-1β) (2). These cytokines directly convert the intrinsic cells in the organs into fibroblasts through receptors on the surface of the cell membrane, leading to the activation of intrinsic cells, producing a large amount of collagen and ECM, and forming the fibrosis (3).

The inflammasomes are intracellular complexes composed of multiple proteins as important components of the innate immune system (4). The inflammasomes are widely expressed in the cytoplasm of the cells, including immune and non-immune cells (5, 6). The immune cells mainly include monocytes (M)/Mø, B cells, T cells, and dendritic cells (DCs) (7–10). The non-immune cells mainly include hepatic stellate cells (HSCs), fibroblasts/myofibroblast (MF), endothelial cells (ECs), and parenchymal cells (PCs) (11–15). The backbone proteins of inflammasomes can recognize the dual signals through pattern recognition receptors (PRRs) on the surface of the cell membrane. The first signal is extracellular pathogen-associated molecular patterns (PAMPs) and the second signal is intracellular damage-associated molecular patterns (DAMPs) (4, 16). The skeleton protein recruits apoptosis-associated speck-like protein containing a CARD (ASC) and pro-cysteinyl aspartate specific proteinase-1 (pro-caspase-1) to form NOD-like receptors (NLRs) and AIM2-like receptors (ALRs) as the main family members of inflammasome complexes (16, 17). The inflammasome complexes induce cells to produce cytokines and cause cell death (4, 16). Cumulative evidences show that the inflammasomes are involved in the fibrogenesis of various organs (18–21). Therefore, it is necessary to elucidate the process of inflammasomes, in particular the canonical pathways for identification of new therapeutic targets for the treatment of fibrosis.

## Classification, Composition and Function of the Inflammasomes

### Classification

According to the activation of cysteinyl aspartate specific proteinase (Caspase) during the formation of inflammasomes, inflammasomes are classified into “classical” and “non-classical” inflammasomes. The “classical” inflammasome mainly activate Caspase-1, while the “non-classical” inflammasome mostly activate other Caspases other than Caspase-1 (22). The “classical” inflammasome involved NOD-like receptors were divided into four classes (NODs, NLRPs, NLRC4 and NLRC5) based on the nucleotide-binding oligomerization domain (NOD, also known as NACHT) (22). (1) NODs, including NOD1-5 and MHC class II transactivator (CIITA). (2) NOD-like receptor proteins (NLRPs), also known as leucine-rich repeat domain proteins (NACHT, LRR and PYD domains-containing proteins, NALPs), including NALP1-14. (3) NOD-like receptor C4 (NLRC4), including IL-1β-converting enzyme-protease-activating factor (IPAF) and neuronal apoptosis inhibitor protein (NAIP). (4) NOD-like receptor C5 (NLRC5), also namely NOD27. In addition, AIM2 belongs to non-NLRs (22). In addition, “non-classical” inflammasome are not clearly classified.

### Composition and Function

**Figure 1:**
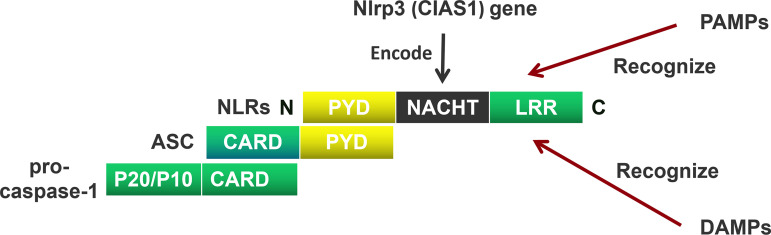
The Composition of the NOD-like receptors (NLRs). NOD-like receptors (NLRs) are mainly composed of a carboxyl (C) terminal, a central terminal, and an amino acid (N) terminal. The C-terminus includes a leucin rich repeat (LRR), the center terminus includes NACHT, and the N-terminus includes a pyrin domain (PYD). The C-terminal LRR recognizes the pathogen-associated molecular patterns (PAMPs) and damage-associated molecular patterns (DAMPs), the central-terminal NACHT encodes by Nlrp3 (CIAS1) gen. The N-terminal CARD interacts with the adaptor protein through PYD-PYD. Apoptosis-associated speck-like protein containing a CARD (ASC) recruits pro-cysteinyl aspartate specific proteinase-1 (pro-caspase-1) through CARD domain to activate downstream signals.

NOD-like receptors (NLRs) are mainly composed of a carboxyl (C) terminal, a central, and an amino acid (N) terminal domain (23). The C-terminus includes a leucin rich repeat (LRR), the center domain includes NACHT, and the N-terminus includes a pyrin domain (PYD) containing a caspase recruitment domain (CARD)/baculoviral inhibitor of apoptosis repeat (BIR)/acidic transactivator (24). The C-terminal LRR recognizes the ligand; the central NACHT hydrolyzes adenosine triphosphate (ATP) by activating a deoxy-ribonucleoside triphosphate (dNTP) enzyme; the N-terminal CARD interacts with the adaptor protein through CARD-CARD to activate downstream signals (22) (**Figure 1**).

The activation of “classical” inflammasome is commonly reported, which usually requires “dual signals” (22). The “first signal” is that the activation signals of toll-like receptors (TLRs), such as *Chlamydia pneumoniae*/*Schistosoma mansoni* (*S. mansoni*), which induce the expression of inflammasomes (25, 26). The “second signal” is composed to the ligands of inflammasomes, such as PAMPs/DAMPs, which induce the activation of inflammasomes. The activation of “classical” inflammasome is mainly that NLRP3 serves as the central skeleton of the inflammasome, and ASC acts as a linker protein connecting NLRP3 with the pro-caspase I, forming inflammasome complexes (4, 16). After activation of the inflammasomes, they depend on Caspase-1 to produce mature IL-1β, IL-18 and IL-33 (22). IL-1β and IL-18 exert biological functions by binding to IL-1/18 receptors (IL-1/18Rs) (27, 28). IL-33 mainly induces Th2 cells to release IL-13 and IL-5 (29). To date, there are few reports on the activation of “non-classical” inflammasome.

The effects of inflammasomes are also generally divided into “classical” and “non-classical” types. The “classical” effect is that the inflammasomes dependent-Caspase-1 induces cells to secrete pro-inflammatory cytokines, removing pathogens and endogenous death signals (30). The “non-classical” effect is that inflammasome components are independent of inflammasome complexes and directly regulate biological processes, such as cell proliferation, gene transcription and translation, and tumor formation (31). The “non-classical” effect of NOD-like receptor protein 3 (NLRP3) is that TGF-β participates in the fibrogenesis through epithelial-mesenchymal transition (EMT) (30).

## The Inflammasomes in the Fibrosis

### NLRP1 Inflammasome

NOD-like receptor protein 1 (NLRP1/NALP1) is called the first inflammasome and exerts its biological activity as an inflammasome complex (32). Toxins and muramyl dipeptide (MDP) as PAMPs lead to the outflow of intracellular potassium ions (K^+^), activating NLRP1 inflammasome and inducing IL-1β secretion by Mø (33).

NLRP1 has been reported to be involved in myocardial fibrogenesis in pressure overload rats (34). NLRP1 mediates myocardial fibrogenesis in mice *via* mitogen-activated protein kinase (MAPK), nuclear factor-κB (NF-κB), and TGF-β/Smad (34). NLRP1 also mediates rat fibrogenesis through TGF-β1/Smad (18). TGF-β1 induces rat cardiac fibroblasts (CFs) to express NLRP1, and through the nuclear translocation of Smad2 and Smad3, promotes the conversion of CFs to MF, leading to ECM deposition and fibrosis (18).

### NLRP3 Inflammasome

#### Activation

NLRP3, also known as NALP3, is usually used as the backbone protein of NLRP3 inflammasome, and forms a complex with ASC and pro-caspase-1, leading to the activation of NLRP3 inflammasome (16).

Currently, there are three hypotheses regarding the activation of NLRP3 inflammasome. (1) K^+^ outflow hypothesis: ATP recognizes P2X7 purinergic receptor (P2X7R) on the cell membrane, opens the ion channel, and leads to K^+^ outflow, recruiting ubiquitinated connexin to punch holes in the cell membrane. The PAMPs enters the cells and promotes the binding of the catalytic domain of NIMA-related kinase 7 (NEK7) to NLRP3 and activates NLRP3 inflammasome (16, 35). (2) Hypothesis of reactive oxygen species (ROS): Streptozotocin (STZ), bleomycin (BLM) and statins first damage mitochondria (36, 37), and then activate phosphatidylinositol 3-kinase (PI3K)/protein kinase B (Akt), c-Jun N-terminal kinase (JNK), and p38/MAPK/extracellular signal-regulated protein kinase (ERK) pathway, respectively, reactivates NADPH oxidase 4 (NOX4), leading to ROS activation (38, 39). ROS induces dissociation of thioredoxin and thioredoxin interacting protein (TXNIP). TXNIP directly activates NLRP3 inflammasome (22). In addition, ROS also induces the conversion of mitochondrial DNA (mtDNA) into oxidized form (ox-mtDNA), which, as the ligand of NLRP3, directly binds and activates NLRP3, activating NLRP3 inflammasome (40). (3) Lysosomal damage hypothesis: the crystals/macromolecules (22, 41), such as beta amyloid, monosodium urate (MSU), airborne particles and cholesterol, activate NADPH oxidase through chemical response, which damages the lysosome, releasing cathepsin B, and activating NLRP3 inflammasome (22, 42). These three hypotheses may explain the activation of NLRP3 inflammasome by some stimulants, but not explain all the activation of NLRP3 inflammasome.

**Figure 2:**
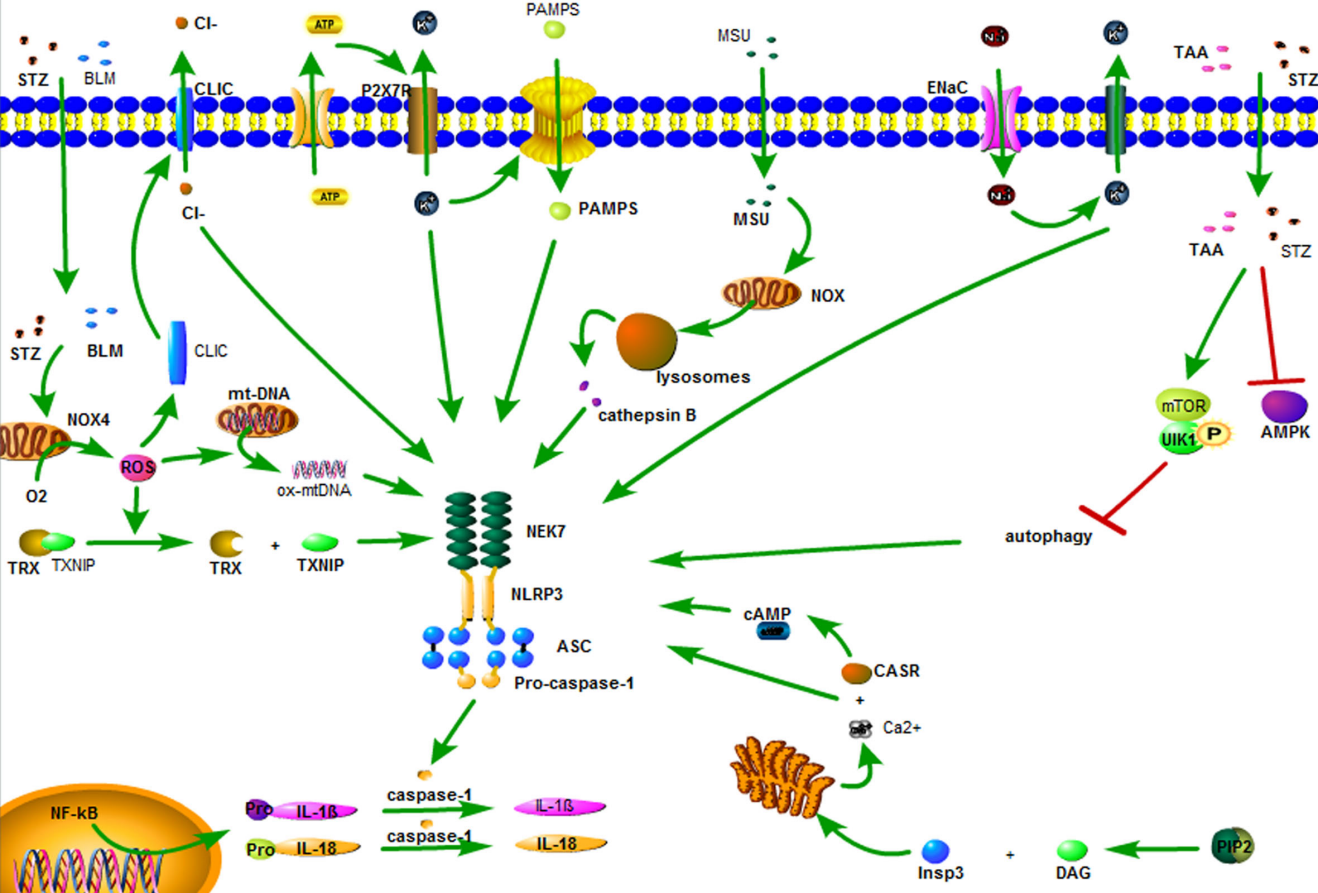
The activation of the NLRP3 inflammasome. ATP recognizes P2X7 purinergic receptor (P2X7R) on the cell membrane, opens the ion channel, and leads to K^+^ outflow, recruiting ubiquitinated connexin to punch holes in the cell membrane. The PAMPs enters the cells and promotes the binding of the catalytic domain of NIMA-related kinase 7 (NEK7) to NLRP3 and activates NLRP3 inflammasome. Streptozotocin (STZ), bleomycin (BLM) and statins first damage mitochondria, and then activate NADPH oxidase 4 (NOX4), leading to ROS activation. ROS induces dissociation of thioredoxin and thioredoxin interacting protein (TXNIP). TXNIP directly activates NLRP3 inflammasome. In addition, ROS also induces the conversion of mitochondrial DNA (mtDNA) into oxidized form (ox-mtDNA), which, as the ligand of NLRP3, directly binds and activates NLRP3, activating NLRP3 inflammasome. The crystals/macromolecules, such as monosodium urate (MSU) activates NADPH oxidase through chemical reaponse, which damages the lysosome, releasing cathepsin B, activating NLRP3 inflammasome. Epithelial sodium channels (ENaC) on the surface of the cell membrane are opened to allow Na^+^ inflow, leading to K^+^ outflow, and activating the NLRP3 inflammasome. Chloride intracellular channels (CLIC) act as the downstream of the K^+^ outflow-mitochondrial ROS axis. ROS induces the transfer of CLIC to the cell membrane, leading to Cl^-^ outflow. The outflow of Cl^-^ enables NEK7 to bind to NLRP3 and promotes the assembly and activation of NLRP3 inflammasome. Phospholipase C hydrolyzes phosphatidylinositol-4,5-diphosphate to form diacyl glycerol (DAG) and inositol trisphosphate (InsP3). InsP3 binds to the InsP3 receptor on the endoplasmic reticulum membrane, causing the endoplasmic reticulum to release Ca^2+^, resulting in an increase in intracellular Ca^2+^. Ca^2+^ is recognized by calcium-sensing receptor (CASR) and activates NLRP3 inflammasome. STZ cooperates with thioacetamide (TAA) through adenosine monophosphate-activated protein kinase (AMPK)/mammalian target of rapamycin (mTOR) pathway inhibition autophagy effect, leading to activation of NLRP3 inflammasome. CASR leads to a decrease in intracellular cAMP, weakens the binding capacity of cAMP and NLRP3, and activates NLRP3 inflammasome.

Aside from the above three hypotheses, there are five ways to activate NLRP3 inflammasome. (1) Sodium ion (Na^+^) inflow: Epithelial sodium channels (ENaC) on the surface of the cell membrane are opened to allow Na^+^ inflow, leading to K^+^ outflow, and activating the NLRP3 inflammasome (43). (2) Chloride (Cl^-^) outflow: chloride intracellular channels (CLIC) act as the downstream of the K^+^ outflow-mitochondrial ROS axis. ROS induces the transfer of CLIC to the cell membrane, leading to Cl^-^ outflow. The outflow of Cl^-^ enables NEK7 to bind to NLRP3 and promotes the assembly and activation of NLRP3 inflammasome (44). (3) Calcium ion (Ca^2+^) accumulation: Phospholipase C hydrolyzes phosphatidylinositol-4,5-diphosphate to form diacyl glycerol (DAG) and inositol trisphosphate (InsP3). InsP3 binds to the InsP3 receptor on the endoplasmic reticulum membrane, causing the endoplasmic reticulum to release Ca^2+^, resulting in an increase in intracellular Ca^2+^. Ca^2+^ is recognized by calcium-sensing receptor (CASR) and activates NLRP3 inflammasome (45). (4) Inhibition of autophagy: STZ cooperates with thioacetamide (TAA) through adenosine monophosphate-activated protein kinase (AMPK)/mammalian target of rapamycin (mTOR) pathway inhibition autophagy effect, leading to activation of NLRP3 inflammasome (46). (5) Cyclic adenosine monophosphate (cAMP) reduction: CASR leads to a decrease in intracellular cAMP, weakens the binding capacity of cAMP and NLRP3, and activates NLRP3 inflammasome (45) (**Figure 2**).

#### Liver Fibrosis

**Figure 3:**
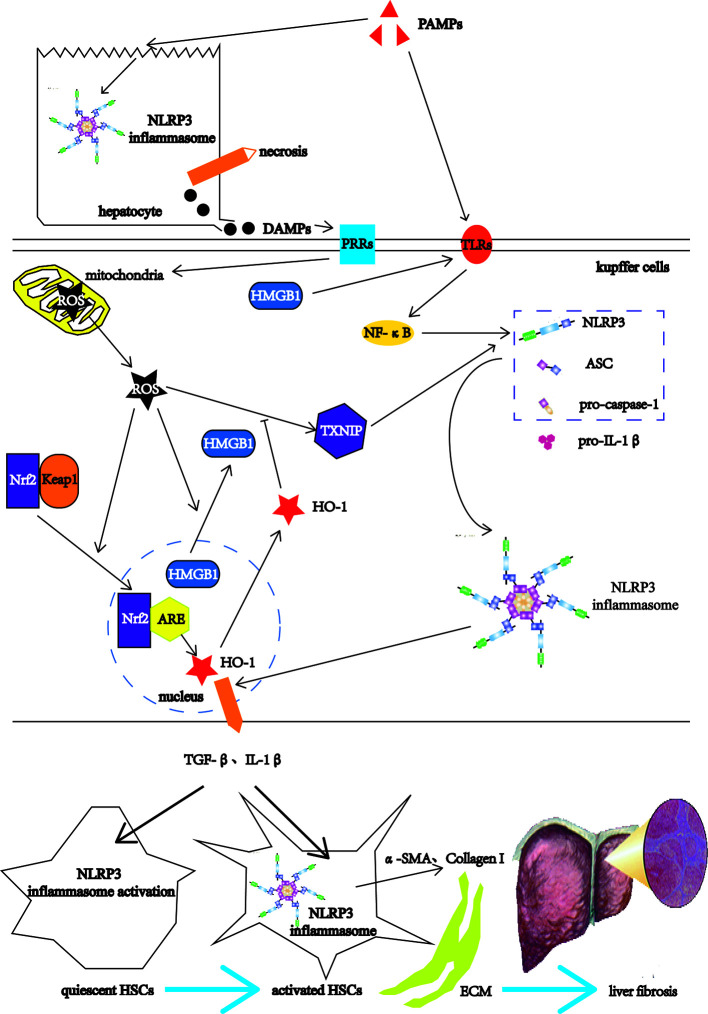
The NLRP3 inflammasome in the liver fibrosis. After the PAMPs act on the livers, they destroy the hepatocytes, and activate the NLRP3 inflammasomes in hepatocytes, leading to hepatocyte necrosis. The necrotic liver cells release DAMPs, which can activate Kupffer cells (KCs). The KCs recognize PAMPs through TLRs on the one hand, and induce the expressions of NLRP3 inflammasome-related pathway components such as NLRP3, pro-caspase-1, and pro-IL-1β through TLRs-NF-κB pathways. On the other hand, the KCs recognize DAMPs, which can directly damage mitochondria and cause them to release ROS. As the upstream signal of NLRP3, ROS activates NLRP3 through the ROS-TXNIP pathway. ROS also promotes the transfer of high mobility group box 1 (HMGB1) from the nucleus to the cytoplasm. The HMGB1 can also activate NLRP3 through the TLR4-NF-κB pathways. After NLRP3 is activated, NLRP3 forms NLRP3 inflammasome together with ASC and pro-caspase-1. Nuclear factor erythroid 2-related factor 2 (Nrf2) is an important transcription factor that regulates cellular anti-oxidative stress. Under physiological conditions, the cytoplasmic protein chaperone molecule Kelch-like ECH-associated protein 1 (Keap1) in KCs binds to Nrf2 and makes it appear to be inhibited. When mitochondria release ROS, Nrf2 dissociates from Keap1 and moves into the nucleus, and combines with the antioxidant response element (ARE) to activate the antioxidant enzyme heme oxygenase-1 (HO-1) expression to inhibit the activation of ROS/NLRP3 inflammasome pathways. The antioxidant response cannot resist the oxidation response, which leads to the KCs activation. Activated KCs activate HSCs by releasing TGF-β and IL-1β, which activate the NLRP3 inflammasome in HSCs. The activated HSCs express α-smooth muscle actin (α-SMA) and Collagen I, leading to ECM deposition and eventually progressing into liver fibrosis.

Liver fibrosis is a common stage of chronic liver injury caused by multiple factors (47). The factors involved in liver fibrogenesis include chemical factors, metabolic factors and infectious factors (48). The chemical factors include ethanol and tetracycline. The metabolic factors include high-fat diet (HFD) and non-alcoholic fatty. The infectious factors include hepatitis B virus (HBV), schistosomes such *S. mansoni* and *S. japonicum* (48). Among them, the infectious factor as PAMPs, after acting on the livers, first destroys the liver cells, the NLRP3 inflammasomes in the liver cells is activated, leading to hepatocyte necrosis (49). The necrotic liver cells release DAMPs, which can activate Kupffer cells (KCs) (11). The KCs recognize PAMPs through TLRs on the one hand, and induce the expressions of NLRP3 inflammasome-related pathway components such as NLRP3, pro-caspase-1, and pro-IL-1β through TLRs-NF-κB pathways (16, 22). On the other hand, the KCs recognize DAMPs, which can directly damage mitochondria and cause them to release ROS (50). As the upstream signal of NLRP3, ROS activates NLRP3 through the ROS-TXNIP pathway (51). ROS also promotes the transfer of high mobility group box 1 (HMGB1) from the nucleus to the cytoplasm (52). As DAMPs, HMGB1 can also activate NLRP3 through the TLR4-NF-κB pathways (53). After NLRP3 is activated, NLRP3 forms NLRP3 inflammasome together with ASC and pro-caspase-1 (54). Nuclear factor erythroid 2-related factor 2 (Nrf2) is an important transcription factor that regulates cellular anti-oxidative stress (55). Under physiological conditions, the cytoplasmic protein chaperone molecule Kelch-like ECH-associated protein 1 (Keap1) in KCs binds to Nrf2 and makes it appear to be inhibited (56). When mitochondria release ROS, Nrf2 dissociates from Keap1 and moves into the nucleus, and combines with the antioxidant response element (ARE) to activate the antioxidant enzyme heme oxygenase-1 (HO-1) expression to inhibit the activation of ROS/NLRP3 inflammasome pathways (57). The antioxidant response cannot resist the oxidation response, which leads to the KCs activation (58). Activated KCs activate HSCs by releasing TGF-β and IL-1β (59). HSCs also have the activation of NLRP3 inflammasome and the self-activation of pro-caspase-1 to form mature Caspase-1 (60). Caspase-1 in HSCs can also catalyze the maturation of pro-IL-1β to form IL-1β and release it outside the cell to form a positive feedback effect (60). Activated KCs recruit monocytes in peripheral blood by releasing CC motif chemokine ligand 2 (CCL2), CCL5, and monocyte chemotactic protein-1 (MCP-1), enlarging inflammatory responses (61, 62). The enlarged inflammatory responses continue to activate HSCs, causing HSCs to express α-smooth muscle actin (α-SMA) and Collagen I, leading to ECM deposition and eventually progressing into liver fibrosis (11) (**Figure 3**).

**Figure 4:**
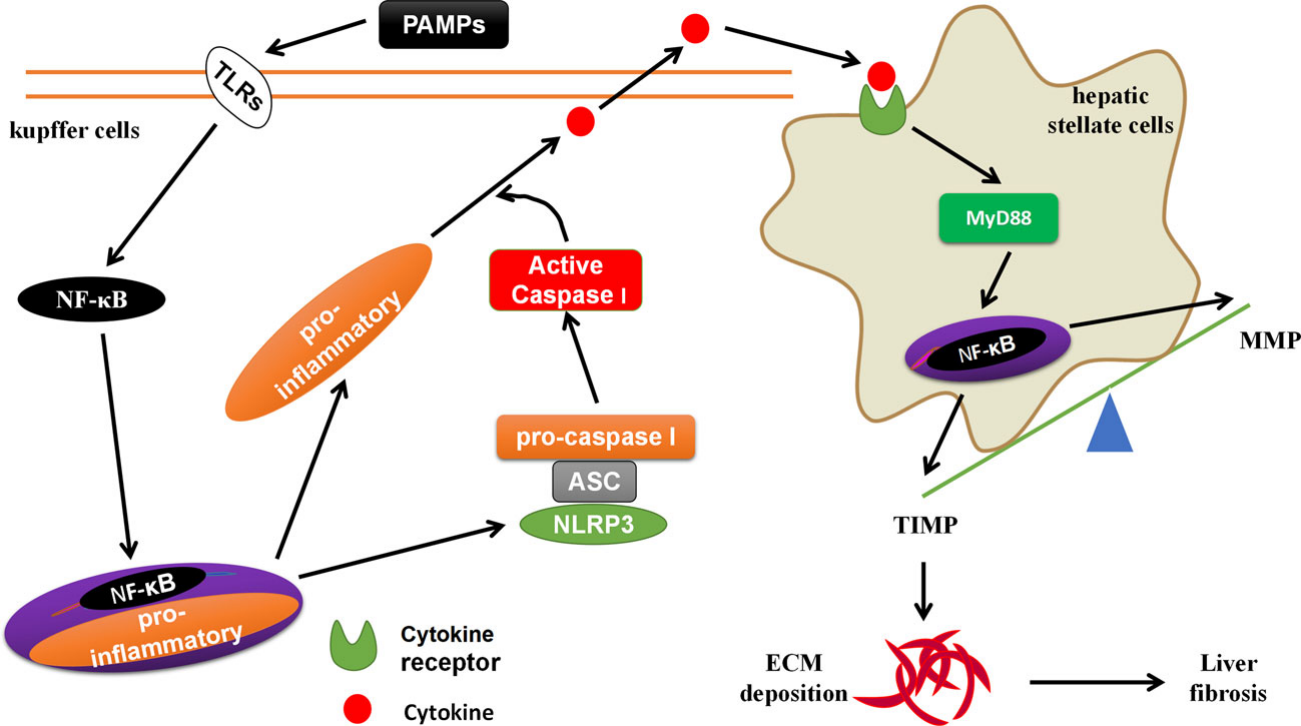
The crosstalk between KCs and HSCs. The PAMPs in the leakage of chronic liver disease can activate NF-κB through TLRs on the surface of KCs, promote the activation of NLRP3 inflammasomes, and induce the generation of pro-inflammatory signals. These pro-inflammatory signals activate HSCs through cytokine receptors (CKRs)/myeloid differentiation factor 88 (MyD88)/NF-κB, leading to liver fibrosis-related molecules matrix metalloproteinases (MMP) and tissue inhibitor of metalloproteinases 1 (TIMP) imbalance, promote ECM deposition and form liver fibrogenesis.

*S. japonicum*, MCD and angiotensin II (Ang II) activate NLRP3 inflammasome through lysosomal damage, inducing oxidative responses (63, 64). The NLRP3 inflammasome mediated Smad3, causes HSCs to express α-SMA, leading to liver fibrosis (19, 65). The formation of liver fibrosis is closely related to the liver-gut axis (66). The PAMPs (such as LPS) in the leakage of chronic liver disease can activate NF-κB through TLRs on the surface of KCs, promote the activation of NLRP3 inflammasomes, and induce the generation of pro-inflammatory signals (such as: IL-1β, IL-18, IL-6, etc.) (67). These pro-inflammatory signals activate HSCs through cytokine receptors (CKRs)/myeloid differentiation factor 88 (MyD88), leading to liver fibrosis-related molecules matrix metalloproteinases (MMP) and tissue inhibitor of metalloproteinases 1 (TIMP) imbalance, promote ECM deposition and form liver fibrogenesis (27, 68) (**Figure 4**). The PAMPs produced by the imbalance of the intestinal flora and the increase in intestinal permeability can be transferred to the liver from the intestine through the bloodstream, which is similar to the effect of PAMPs from chronic liver diseases (69). MCC950, an inhibitor of NLRP3 inflammasome activation, could block the activation of NLRP3 inflammasome, reduce the production of TGF-β and Collagen I, and delay progression of liver fibrogenesis (64, 70). However, systemic knock-in of *NLRP3* gene in mice accelerates the progression of liver fibrogenesis by promoting the activation of NLRP3 inflammasome, inducing hepatocyte pyrolysis, forming severe liver tissue inflammation (49). The hepatocytes can also directly participate in liver fibrogenesis (71). Professor Li et al. reported that Ang II generates hydrogen peroxide (H_2_O_2_) through NOX4 by acting on the angiotensin II type-1 receptor (AT1R) on the surface of hepatocytes (71). The H_2_O_2_ activates the NLRP3 inflammasome to produce IL-1β. IL-1β induces the phosphorylation of Smad2/3 to promote the transformation of hepatocytes to EMT, expressing Collagen I, and forming liver fibrosis (71).

Hepatic stellate cells (HSCs), as a key cell type of the liver, are involved in the development of liver fibrogenesis by HSCs activation (11). The soluble egg antigen (SEA) of *Schistosoma japonicum* activates the NLRP3 inflammasome in HSCs by activating spleen tyrosine kinase (Syk), C-type lectin receptor Dectin-1 and JNK pathway (72). Ang II up-regulates mir-21 expression by targeting Smad7 and Spry1. On the one hand, mir-21 inhibits Smad7 by targeting and releases the inhibitory effect of Smad7 on Smad2/3, leading to the activation of Smad2/3/NOX4/ROS (38). On the other hand, mir-21 inhibits Spry1 by targeting and releases releases the inhibitory effect of Spry1 on ERK, which activates the ERK/NF-κB signaling pathway, leading to the activation of NLRP3 inflammasomes (38). After the mouse primary HSCs or hepatic stellate cell lines (LX-2/HSC-T6) are treated with exogenous stimulants, such as MSU/bacterial RNA, NLRP3 inflammasome can be activated and induce HSCs to secrete IL-1β (70, 73). IL-1β interacts with IL-1Rs on the membrane of HSCs, which activates NF-κB and causes TGF-β expression. TGF-β induces the expression of α-SMA and Collagen I through the TGF-βR on the cell membrane of HSCs (70, 73). In addition to IL-1β and TGF-β, tumor necrosis factor (TNF) and IL-17 also play a role in the comparable way (74).

Most views suggest that NLRP3 inflammasome participates in liver fibrogenesis in an “indirect” manner with the activation of other signals (3, 38, 74). However, there have been reported that NLRP3 inflammasome is independent of cytokines, and is directly expressed and activated in HSCs, and is involved in liver fibrogenesis in a “direct” manner with HSCs activation (75). Basing on above reports, the “indirect” and “direct” manners are coexist, and the “indirect” manner plays a major role in liver fibrogenesis.

#### Cardiac Fibrosis

Fructose activates NLRP3 inflammasome by inducing ROS production (75). NLRP3 inflammasome reactivates Smad2/3, leading to cardiac fibrosis (76). CFs are the key cells of cardiac fibrosis and are involved in the development of cardiac fibrogenesis (77). After TGF-β was administered to mouse primary CFs, CFs differentiated significantly (75). After the primary CFs of NLRP3^-/-^ mice were isolated and were added TGF-β, the differentiation of CFs is weakened (76). The results suggest that NLRP3 plays an important role in the differentiation of CFs. In addition to NLRP3 participating in the differentiation of CFs as a single molecule, it also participates in cardiac fibrogenesis *via* the activation of NLRP3 inflammasome and the product IL-1β (78). IL-1β plays a role in promoting cardiac fibrogenesis through the conversion of CFs to MF (79).

#### Lung Fibrosis

Lipopolysaccharide (LPS) and BLM activate NLRP3 inflammasome *via* ROS (80, 81). NLRP3 inflammasome lead to pulmonary fibrosis through the IL-1β/IL-1Rs/MyD88/NF-κB signaling pathway (81). Lung fibroblasts are key cells for pulmonary fibrosis (82). After isolation of mouse primary lung fibroblasts, BLM was added to the mouse primary lung fibroblasts, and it was found that NLRP3 inflammasome regulate IL-1β *via* miR-155, leading to lung fibrosis (83, 84). It can be seen that IL-1β plays an important role in the formation of pulmonary fibrogenesis. In addition to IL-1β, TGF-β and platelet derived growth factor-AA (PDGF-AA) have the comparable function (84). In recent years, lung ECs have been the focus of research on pulmonary fibrogenesis. The studies have found that NLRP3 inflammasome transforms ECs into EMT, forming pulmonary fibrosis (85).

#### Renal Fibrosis

Adenine diet and unilateral ureteral obstruction (UUO) can both induce NLRP3 inflammasome activation *via* ROS (86, 87). Recently, there have also been reports of NLRP3 inflammasome-dependent NF-κB activation after major nephrectomy (88). NLRP3 inflammasome activates the T cells and induces renal fibrogenesis through the IL-23/IL-17 axis (86). Recently, the report showed that MCC950 was given too late to sufficiently block renal inflammation, and not to delaying the progression of renal fibrogenesis (87).

Endothelial cells (ECs) have also received attention in renal fibrogenesis. After TGF-β was administered to tubular epithelial cells (TECs) in mice, NLRP3 expression was increased and NLRP3 transformed TECs into EMT, and then into MF through the phosphorylation of Smad2/3, resulting in increased expression of α-SMA and matrix metalloprotein 9 (MMP9). After TGF-β was given to the primary TECs of NLPR3^-/-^mice, the NLRP3 expression was decreased, the phosphorylation of Smad2/3 was decreased, and the expression of α-SMA and MMP9 was decreased (14). The above reports display that NLRP3 promotes the conversion of TECs to renal fibrosis through the TGF-β/Smad pathway.

#### Other Fibrosis

NLRP3 inflammasome is also involved in pancreatic fibrogenesis caused by bombesin, peritoneal fibrogenesis caused by methylglyoxal (MGO), cystic fibrogenesis caused by *Aspergillus fumigatus* (*A. fumigatus*)/*Pseudomonas aeruginosa* (*P. aeruginosa*), and bladder fibrogenesis caused by bladder opening obstruction (27, 89–91). Using the same NLPR3^-/-^ mice, it was found that peritoneal fibrosis was reduced after MGO was administration (90). The reports demonstrate that the NLRP3 is a key molecule in fibrogenesis.

### NLRC4 Inflammasome

The NLRC4 inflammasome is usually activated by the flagellin of gram-positive and gram-negative bacteria and endolin of type III secretion system (T3SS) derived from gram-negative bacteria (92, 93). For example, NAIP5 in mice is activated by bacterial flagellin, while NAIP in humans is activated by the needle-like subunits of T3SS (92, 93). However, the activation mechanisms are unclear. In *A. fumigatus* or *P. aeruginosa* infected mice, NLRC4 expression depended on cystic fibrosis transmembrane conductance regulator (CFTR) reached a peak at 7 days (27). However, it has also been reported that NLRC4 produces IL-1R antagonist (IL-1Ra) *via* NF-κB, to bind IL-1β, delaying the progression of fibrogenesis (27). Furthermore, NLRC4 has also been reported to promote liver cell regeneration and reverse liver fibrosis (20). But the molecular mechanism in which NLRC4 plays a negative role in fibrogenesis remains to be studied.

### AIM2 Inflammasome

AIM2 inflammasome is composed of oligosaccharides and PYD domains (17). AIM2 inflammasome recognizes the double-stranded DNA (dsDNA) in the cell through the oligosaccharide domain, and then bind to ASC through the PYD domain, leading to pro-caspase-1 self-activation (17). After Peripheral blood mononuclear cells (PBMCs) were treated with Poly (dA: dT), the expression of AIM2 inflammasome was increased (21). AIM2 inflammasome dependent on Caspase-4 induces IL-1α secretion by PBMCs. IL-1α binds to IL-1αR, inducing TGF-β secretion by PBMCs (21). TGF-β is a key factor in the fibrogenesis (94). It can be concluded that AIM2 inflammasome participates in the development of fibrogenesis.

### Other Inflammasomes

NOD-like receptor protein 6 (NLRP6) is mainly expressed in the small intestine, large intestine, and liver (95). NLRP6, as a special functional protein in the NLRs family, has a “negatively regulation” to liver fibrogenesis (96, 97). *In vivo* experiments found that in allogeneic hematopoietic stem cell transplantation (Allo-HSCT) mice, NLRP6 inhibits liver fibrogenesis through the activation of p38/MAPK, NF-κB and NLRP3 inflammasome, respectively (96). *In vitro* experiments found that NLRP6 inhibited the proliferation and activation of LX-2 cells, and by enhancing the expression of magnesium ion-dependent protein phosphatase 1A (PPM1A), it inhibited the phosphorylation of Smad2/3 and reduced the expression of Collagen I and Collagen III (97).

NOD-like receptor C5 (NLRC5) belongs to the largest member of the NLRs family and is expressed in the cytoplasm and nucleus of most cells (98). NLRC5 is also involved in the development of fibrogenesis. NLRC5 expression is present in liver tissues of patients with cirrhosis and also found in CCl_4_ treated mice (99, 100). In addition, TGF-β regulates Smad2/3 and NF-κB *via* NLRC5, induces LX-2 activation and expresses α-SMA and Collagen I (99, 100).

## The Inflammasome-Associated Molecules in the Fibrosis

### Caspase-1 in Fibrosis

Caspase-1 is mainly used as the activation product of “classical” inflammasome and is involved in the fibrogenesis (101). Caspase-1 catalyzes maturation of pro-IL-1β and secretion of IL-1β (22). IL-1β has a pro-fibrotic effect, and is usually involved in the fibrogenesis in combination with IL-1βRs on the surface of resident cells (102). In BLM-induced pulmonary fibrogenesis mice, the inhibitor of Caspase-1, YVAD-fmk, delays the progression of pulmonary fibrogenesis (81). Once the production of caspase-1 was blocked by YVAD-fmk, the interaction between NLRP3 and ASC, ASC and pro-caspase-1 was weakened (81). It suggested that YVAD-fmk inhibits the production of caspase-1, hinders the formation of NLRP3 inflammasome, and delays the progression of pulmonary fibrogenesis. Similar reports have been displayed in *S. japonicum* infection and HFD-induced liver fibrogenesis (63, 103).

### IL-1β in Fibrosis

IL-1β is mainly secreted by activated M, Mø, and DCs (104). At present, there are three main types of IL-1β secretion mechanisms. (1) ATP causes K^+^ outflow and Ca^2+^ inflow, then activated phospholipase C and phospholipase A2, resuting in cells to secrete IL-1β (22). (2) IL-1β secretion after inflammasome formation and activation (27). (3) The Caspase-4 and Caspase-1 are activated sequentially, and induce PBMCs to secrete IL-1β (105). IL-1β binds to IL-1Rs on the surface of cell membranes, and promotes pro-IL-1β transcription and translation to produce IL-1β (22). IL-1β could also promote hepatocyte apoptosis, activate M and neutrophils, leading to fibrosis (106).

As a key signal for leading to fibrogenesis, IL-1β plays a role in promoting fibrogenesis by binding to IL-1βRs (102). IL-1β and IL-1βRs are in a dynamic equilibrium. The agonists of IL-1βRs can promote fibrogenesis through IL-1β (107), but the antagonists of which can prevent the fibrogenesis of IL-1β promotion by reducing IL-1βRs (27). IL-1β promotes fibrogenesis through TGF-β, ERK1/2, c-Jun, and PI3K/Akt, respectively (108). IL-1β also promotes renal stromal cells (SCs) through the IL-1 receptors-IL-1R-related kinase 4 (IRAK4) -protocogene (MYC) transcription factor axis, to expresses platelet-derived growth factor receptor (PDGFR) (102). PDGFR, in combination with PDGF, promotes the appreciation and migration of SCs, leading to the deposition of ECM and the formation of renal fibrosis (109).

### IL-18 in Fibrosis

IL-18, also known as interferon-γ inducing factor (IGIF), is usually expressed in a variety of cells as pro-IL-18 (110). In addition to being cleaved by “classical” Caspase-1, IL-18 is also cleaved by “non-classical” protease 3/Caspase-3 (111, 112). Pro-IL-18 is cleaved into mature IL-18 and secreted extracellularly by the cells (4). Most studies report that IL-18 has a profibrotic effect. There are currently three main ways to promote fibrogenesis. (1) IL-18 induces Th1 cells produce IFN-γ and IL-13, causing fibrosis (113). (2) Ischemia-reperfusion injury induces Mø to M2-type cells through IL-18, forming fibrosis (114). (3) IL-18 recruits T cells and Mø *via* chemokines, and transforms Mø into MF, resulting in fibrosis (115). After administration of IL-18 inhibitors, T cells and Mø decreased and the transformation of Mø into MF slowed (114).

A few publications report that IL-18 has an anti-fibrotic effect. The expression of IL-18 in serum and liver tissue was induced by DNA vaccine of IL-18, reducing schistosome-associated liver fibrosis (SSLF) (116). IL-18 was transfected into *S. japonicum*-infected hepatocytes, and hepatocytes expressed IL-18. IL-18 reverses the conversion of Th1 to Th2, improving SSLF (117). The anti-fibrotic effect of IL-18 mainly occurs in SSLF, and it may be related to the pathogenic way of *S. japonicum*. Whether IL-18 exerts the effect of promoting fibrogenesis or suppressing fibrogenesis remains to be proved experimentally.

### IL-33 in Fibrosis

IL-33, also known as the 11th member of the IL-1 family (IL-1F11), is usually expressed in the nucleus of ECs, fibroblasts and immune cells in the form of pro-IL-33 (118). When cell death or tissue damage occurs, pro-IL-33 is cleaved by Caspase-1/Caspase-3/Caspase-7 to mature IL-33 (118). IL-33 is secreted outside the cell as an “alarmin” and participates in tissue homeostasis by Th2 cells (29).

IL-33 promotes fibrogenesis in two main ways. (1) Pro-fibrosis effect of IL-33/ST2 signal axis: In liver fibrogenesis caused by *S. japonicum* infected with mice, the expressions of IL-33 and ST2 in the liver are increased (119). ST2 is a ligand of IL-33 (114). IL-33 is dependent on ST2 for MCD diet-induced liver fibrogenesis (120). In addition, IL-33/ST2 is also involved in BLM and *E. coli*-induced fibrogenesis (121). In BLM-induced fibrogenesis, IL-33/ST2 through B7 homology 3 (B7H3), polarizes bone marrow (BM) cells to M2 cells, secreting IL-13 and TGF-β (122, 123). (2) IL-33 cooperated with other molecules to promote fibrogenesis: IL-33, IL-25 and thymic stromal lymphopoietin (TSLP) involved in secondary pulmonary fibrogenesis caused by *S. mansoni* (124).

*In vitro* experiments showed that *P. aeruginosa* induced the IL-33 expression in the cystic fibrosis airway epithelial cell line (CFTRdelF508) (125). IL-33 promotes human primary eosinophils to express IL-13; IL-13 induces the MF in the intestine to express Collagen (126). The renal tubular cell line (HK-2) was pretreated with IL-33shRNA, then treated with hypoxia and reoxygenation, and α-SMA and Collagen I expressions were reduction (127).

## Summary and Future Direction

There are many reports about the role of NLRP3 inflammasome in the fibrogenesis, but there is insufficient evidence on how NLRP3 inflammasome regulate fibrogenesis. NLRP1 and AIM2 inflammasomes are rarely studied in the fibrogenesis and need to understand the phenomenon from the molecular mechanism. More and more studies suggest it is very important that NLRC4 inflammasome in the fibrogenesis, but the molecular mechanism remains to be experimentally elucidated. The role of other inflammasomes in the fibrogenesis has also been reported, such as the role of NLRP6/NLRC5 in the fibrogenesis. IL-1β, IL-18, and IL-33, as activation products of inflammasomes, usually participate in fibrogenesis with other signaling pathways. However, the effect of IL-18 on the fibrogenesis is still controversial, and more experiments are needed to determine whether IL-18 promotes fibrogenesis or inhibits fibrogenesis. In summary, the mechanism of inflammasomes is not completely clear, and the relationship with fibrogenesis deserve more in-depth investigations. The solution of these investigations helps to clarify the role of inflammasomes in fibrogenesis and find new targets for the treatment of fibrosis. Through these targets, drugs focusing on inflammasome-associated molecules are developed to treat fibrotic diseases. Therefore, more in-depth researches centering on inflammasome and fibrosis are necessary.

## Author Contributions

W-JZ, HW and S-ZW conceived, performed and designed the topics. W-JZ gathered and read papers, as well as wrote the first draft of the manuscript. S-JC, HW and S-ZW corrected and validated the manuscript. S-JC drew the figures. All authors contributed to the article and approved the submitted version.

## Funding

This work was financially supported by the grants from The Open Project of Key Laboratory of Prevention and treatment of cardiovascular and cerebrovascular diseases, Ministry of Education (No. XN202016).

## Conflict of Interest

The authors declare that the research was conducted in the absence of any commercial or financial relationships that could be construed as a potential conflict of interest.

## Abbreviations

NLRP1, NOD-like receptor protein 1; NLRP3, NOD-like receptor protein 3; NLRC4, NOD-like receptor C4; AIM2, absent in melanoma 2; ECM, extracellular matrix; mø, macrophages; TGF-β, transforming growth factor-β; IL-1β, interleukin-1β; M, monocytes; DCs, dendritic cells; HSCs, hepatic stellate cells; MF, myofibroblast; ECs, endothelial cells; PCs, parenchymal cells; PRRs, pattern recognition receptor; PAMPs, pathogen-associated molecular patterns; DAMPs, damage-associated molecular patterns; ASC, apoptosis-associated speck-like protein containing a CARD; pro-caspase-1, pro-cysteinyl aspartate specific proteinase-1; NLRs, NOD-like receptors; ALRs, AIM2-like receptors; Caspase, cysteinyl aspartate specific proteinase; NOD, nucleotide-binding oligomerization domain; NLRPs, NOD-like receptor proteins; NALPs, NACHT, LRR and PYD domains-containing proteins; IPAF, IL-1β-converting enzyme-protease-activating factor; NAIP, neuronal apoptosis inhibitor protein; NLRC5, NOD-like receptor C5; LRR, leucin rich repeat; CARD, caspase recruitment domain; PYD, pyrin domain; BIR, baculoviral inhibitor of apoptosis repeat; dNTP, deoxy-ribonucleoside triphosphate; ATP, adenosine triphosphate; *C. pneumoniae*, *Chlamydia pneumoniae*; *S. mansoni, Schistosoma mansoni*; IL-1/18Rs, interleukin-1/18 receptors; EMT, epithelial-mesenchymal transition; MDP, muramyl dipeptide; MAPK, mitogen-activated protein kinase; NF-κB, nuclear factor-κB; CFs, cardiac fibroblasts; P2X7R, P2X7 purinergic receptor; NEK7, NIMA-related kinase 7; ROS, reactive oxygen species; STZ, streptozotocin; BLM, bleomycin; PI3K, phosphatidylinositol 3-kinase; Akt, protein kinase B; JNK, c-Jun N-terminal kinase; ERK, extracellular signal-regulated protein kinase; NOX4, NADPH oxidase 4; TXNIP, thioredoxin interacting protein; mtDNA, mitochondrial DNA; ox-mtDNA, oxidized form; MSU, monosodium urate; ENaC, epithelial sodium channels; CLIC, chloride intracellular channels; DAG, diacyl glycerol; InsP3, inositol trisphosphate; CASR, calcium-sensing receptor; TAA, thioacetamide; AMPK, adenosine monophosphate-activated protein kinase; mTOR, mammalian target of rapamycin; cAMP, cyclic AMP; CCl_4_, carbon tetrachloride; DMN, dimethylnitrosamine; DEN, diethylnitrosamine; HFD, high-fat diet; MCD, methionine/choline-deficient diet; HBV, hepatitis B virus; *S. japonicum, Schistosoma japonicum*; KCs, kupffer cells; CCL2, CC motif chemokine ligand 2; α-SMA, α-smooth muscle actin; *E. coli*, *Escherichia coli*; TNF, tumor necrosis factor; LPS, lipopolysaccharide; PDGF-AA, platelet derived growth factor-AA; UUO, unilateral ureteral obstruction; TECs, tubular epithelial cells; MMP9, matrix metalloprotein 9; MGO, methylglyoxal; *A. fumigatus*, *Aspergillus fumigatus*; *P. aeruginosa*, *Pseudomonas aeruginosa*; T3SS, type III secretion system; CFTR, cystic fibrosis transmembrane conductance regulator; IL-1Ra, IL-1R antagonist; dsDNA, double-stranded DNA; PBMCs, peripheral blood mononuclear cells; DENV, dengue virus; IRAK4, IL-1 receptor-associated kinase 4; SCs, stromal cells; PDGFR, platelet-derived growth factor receptor; IGIF, interferon-γ inducing factor; SSLF, schistosome-associated liver fibrosis; IL-1F11, interleukin-1 family 11; B7H3, B7 homology 3; BM, bone marrow; TSLP, thymic stromal lymphopoietin; NLRP6, NOD-like receptor protein 6; Allo-HSCT, allogeneic hematopoietic stem cell transplantation.

## Glossary

**Table:**

NLRP1	NOD-like receptor protein 1
NLRP3	NOD-like receptor protein 3
NLRC4	NOD-like receptor C4
AIM2	absent in melanoma 2
ECM	extracellular matrix
mø	macrophages
TGF-β	transforming growth factor-β
IL-1β	interleukin-1β
M	monocytes
DCs	dendritic cells
HSCs	hepatic stellate cells
MF	myofibroblast
ECs	endothelial cells
PCs	parenchymal cells
PRRs	pattern recognition receptor
PAMPs	pathogen-associated molecular patterns
DAMPs	damage-associated molecular patterns
ASC	apoptosis-associated speck-like protein containing a CARD
pro-caspase-1	pro-cysteinyl aspartate specific proteinase-1
NLRs	NOD-like receptors
ALRs	AIM2-like receptors
Caspase	cysteinyl aspartate specific proteinase
NOD	nucleotide-binding oligomerization domain
NLRPs	NOD-like receptor proteins
NALPs, NACHT	LRR and PYD domains-containing proteins
IPAF	IL-1β-converting enzyme-protease-activating factor
NAIP	neuronal apoptosis inhibitor protein
NLRC5	NOD-like receptor C5
LRR	leucin rich repeat
CARD	caspase recruitment domain
PYD	pyrin domain
BIR	baculoviral inhibitor of apoptosis repeat
dNTP	deoxy-ribonucleoside triphosphate
ATP	adenosine triphosphate
*C. pneumoniae*	*Chlamydia pneumoniae*
*S. mansoni*	*Schistosoma mansoni*
IL-1/18Rs	interleukin-1/18 receptors
EMT	epithelial-mesenchymal transition
MDP	muramyl dipeptide
MAPK	mitogen-activated protein kinase
NF-κB	nuclear factor-κB
CFs	cardiac fibroblasts
P2X7R	P2X7 purinergic receptor
NEK7	NIMA-related kinase 7
ROS	reactive oxygen species
STZ	streptozotocin
BLM	bleomycin
PI3K	phosphatidylinositol 3-kinase
Akt	protein kinase B
JNK	c-Jun N-terminal kinase
ERK	extracellular signal-regulated protein kinase
NOX4	NADPH oxidase 4
TXNIP	thioredoxin interacting protein
mtDNA	mitochondrial DNA
ox-mtDNA	oxidized form
MSU	monosodium urate
ENaC	epithelial sodium channels
CLIC	chloride intracellular channels
DAG	diacyl glycerol
InsP3	inositol trisphosphate
CASR	calcium-sensing receptor
TAA	thioacetamide
AMPK	adenosine monophosphate-activated protein kinase
mTOR	mammalian target of rapamycin
cAMP	cyclic AMP
CCl_4_	carbon tetrachloride
DMN	dimethylnitrosamine
DEN	diethylnitrosamine
HFD	high-fat diet
MCD	methionine/choline-deficient diet
HBV	hepatitis B virus
*S. japonicum*	*Schistosoma japonicum*
KCs	kupffer cells
CCL2	CC motif chemokine ligand 2
α-SMA	α-smooth muscle actin
*E. coli*	*Escherichia coli*
TNF	tumor necrosis factor
LPS	lipopolysaccharide
PDGF-AA	platelet derived growth factor-AA
UUO	unilateral ureteral obstruction
TECs	tubular epithelial cells
MMP9	matrix metalloprotein 9
MGO	methylglyoxal
*A. fumigatus*	*Aspergillus fumigatus*
*P. aeruginosa*	*Pseudomonas aeruginosa*
T3SS	type III secretion system
CFTR	cystic fibrosis transmembrane conductance regulator
IL-1Ra	IL-1R antagonist
dsDNA	double-stranded DNA
PBMCs	peripheral blood mononuclear cells
DENV	dengue virus
IRAK4	IL-1 receptor-associated kinase 4
SCs	stromal cells
PDGFR	platelet-derived growth factor receptor
IGIF	interferon-γ inducing factor
SSLF	schistosome-associated liver fibrosis
IL-1F11	interleukin-1 family 11
B7H3	B7 homology 3
BM	bone marrow
TSLP	thymic stromal lymphopoietin
NLRP6	NOD-like receptor protein 6
Allo-HSCT	allogeneic hematopoietic stem cell transplantation.
